# Dynamic 3D shape of the plantar surface of the foot using coded structured light: a technical report

**DOI:** 10.1186/1757-1146-7-5

**Published:** 2014-01-23

**Authors:** Ali K Thabet, Emanuele Trucco, Joaquim Salvi, Weijie Wang, Rami J Abboud

**Affiliations:** 1 Institute of Motion Analysis and Research, Department of Orthopedic and Trauma Surgery, Tayside Orthopedics and Rehabilitation Technology Centre, Ninewells Hospital and Medical School, University of Dundee, Dundee, DD1 9SY, UK; 2 School of Computing, University of Dundee, Dundee, DD1 4HN, UK; 3 Computer Vision and Robotics Group, University of Girona, 17071 Girona, Spain

**Keywords:** Biomechanics, 3D foot reconstruction, Dynamic foot, Foot measurements, CAD

## Abstract

**Background:**

The foot provides a crucial contribution to the balance and stability of the musculoskeletal system, and accurate foot measurements are important in applications such as designing custom insoles/footwear. With better understanding of the dynamic behavior of the foot, dynamic foot reconstruction techniques are surfacing as useful ways to properly measure the shape of the foot. This paper presents a novel design and implementation of a structured-light prototype system providing dense three dimensional (3D) measurements of the foot in motion. The input to the system is a video sequence of a foot during a single step; the output is a 3D reconstruction of the plantar surface of the foot for each frame of the input.

**Methods:**

Engineering and clinical tests were carried out to test the accuracy and repeatability of the system. Accuracy experiments involved imaging a planar surface from different orientations and elevations and measuring the fitting errors of the data to a plane. Repeatability experiments were done using reconstructions from 27 different subjects, where for each one both right and left feet were reconstructed in static and dynamic conditions over two different days.

**Results:**

The static accuracy of the system was found to be 0.3 mm with planar test objects. In tests with real feet, the system proved repeatable, with reconstruction differences between trials one week apart averaging 2.4 mm (static case) and 2.8 mm (dynamic case).

**Conclusion:**

The results obtained in the experiments show positive accuracy and repeatability results when compared to current literature. The design also shows to be superior to the systems available in the literature in several factors. Further studies need to be done to quantify the reliability of the system in clinical environments.

## Background

The foot provides a crucial contribution to the balance and stability of the musculoskeletal system [1], and accurate foot measurements are important in applications such as designing custom insoles/footwear. In recent years, digitally acquired scans of the foot have gained popularity among clinicians and foot specialists [2]. Commercially available foot scanners provide static 3D reconstructions of the foot, but due to their expensive nature (prices range from 5000 to 50000 USD), different research systems have investigated more cost-efficient solutions [3-7]; a good summary of 3D foot scanning methods can be found in the work by Telfer *et al.*[8].

With increasing understanding of the dynamic behavior of the foot, research is pointing towards the use of dynamic foot models as better descriptors for foot measurements [9-11]. Several dynamic 3D reconstruction systems are available in the literature. In order to assess the efficiency and usability of such systems, two factors are of essence, design and implementation, and clinical usability. In terms of the former, all systems acquire foot shape using a variation of *stereovision*, a technique that requires imaging a surface from at least two different views in order to reconstruct its 3D shape [12]. The different views usually come from a combination of cameras and projectors. Assessment in this area is usually done by looking at the amount of equipment needed, as well as its reconstruction accuracy and repeatability. When it comes to clinical usability, a primary intent should be to acquire the reconstructions without involving the addition of artifacts to the subjects foot, which could be in the form of markers, socks, paint, and others; abstaining from these additions will ensure the most natural walking patterns possible and therefore provide reconstructions that better reflect the subjects dynamic foot behavior. A second important point to consider is the repeatability of the system under clinical trials, to that extent, any design must be able to provide equally reliable measures of the same foot at different recording times.

One of the earliest dynamic reconstruction systems was designed by Codert *et al.*, to reconstruct the surface of the entire foot during the gait cycle [13]. The system was composed of 6 cameras that worked in a synchronized manner. As with most camera based reconstruction systems, lack of texture in the target surface, the foot in this case, limits the performance of the reconstruction. The authors considered two ways of overcoming this obstacle; one was to cover the foot with a sock while the second involved spraying the foot surface with paint in order to embed a random grey level pattern on it. These methods worked well during the reconstruction process as Codert *et al.* showed in their visual results. Although this technique provided dynamic foot reconstructions, it involved adding an extra factor to the foot surface (either socks or paint), something that is undesirable in a clinical environment.

In 2009, Jezersek and Mozina developed a high-speed foot measurement system using multiple laser-camera pairs [14]. The main motivation behind their work was to provide static foot reconstructions at high frame rates. The presence of the laser units helps adding texture to the foot without involving external artifacts on its surface; a technique commonly referred to a *structured lighting*[15]. Due to the intent of reconstructing the whole foot surface, the system also had mirrors at places where the cameras had no visual reach, and therefore making the system design more complex. Given the high-speed nature of their system, the authors briefly mention a possible application to dynamic foot reconstruction. Although the systems proves accurate and repeatable in static conditions, little is mentioned on its abilities during dynamic situations. This work was further studied in 2011 to focus more on dynamic reconstructions [16].

A dynamic foot cross-section measurement technique was presented in 2009 by Kouchi *et al.*[17]. The system is composed of 12 synchronized high-resolution cameras, positioned in pairs in order to cover the whole surface of the foot. The author’s main interest is to measure cross-section profiles of the foot, in particular the areas defined as Forefoot, Instep, Navicular, and Heel cross-sections. In order to properly recognize the areas of interest in the captured images, 4 profile lines are drawn on the foot surface, allowing tracking of foot sections over time, therefore providing 4D measurements. Because of the crucial need of highlighting the cross-section lines on the foot, the system cannot be used to get dense foot reconstructions (reconstructions where the acquired 3D points a closely packed, therefore providing surface data at high resolutions). The painting of artifacts of the surface of the foot might also be undesirable in clinical scenarios.

Schmeltzpfenning *et al.* considered a structured light approach to reconstruct the plantar surface of the foot [18], and the whole surface of the foot [19]. The presented system uses 3 camera-projector pairs, which work in an interleaved manner. Although the authors use the system to measure changes in foot shape during the stance phase of walking, there is no clear analysis on the accuracy of the system measurements and it is therefore difficult to assess the reliability of their measurements.

Mochimaru and Kouchi presented a single camera/projector system to reconstruct the plantar surface of the foot in motion [20]. The projector illuminates the foot with small squares of randomly colored patterns, a factor that can make the processing of the image data computationally expensive, and provide larger number of reconstruction errors. This work was further developed by Yoshida and Kouchi [21], where the remainder of the foot surface was estimated by adding 3 more camera-projector units. With the whole foot surface available, the new system uses a previously computed and densely reconstructed generic foot model to enhance the 3D reconstructions of the foot. The idea behind this approach is that any reconstructed foot model can be modeled as a variation of the generic foot; such an assumption will make the full surface reconstruction process more efficient in terms of computational time. There are two major flaws with this final approach. First, the model used for fitting was obtained from a static reconstruction, and therefore fitting it to deformed foot reconstructions might not provide accurate results; the authors do not provide any measure of accurate dynamic reconstruction to validate their fitting hypothesis. Second, it is unclear how the system will behave in the presence of a deformed foot; severe deformities may prove to be significantly different to the generic foot model shape and the optimization thus may not converge.

Liu *et al.* presented a dynamic foot scanner using time-of-flight cameras [22]. The reconstruction system can measure the entire surface of the foot at high speed and therefore provide full dynamic reconstructions. The 3 cameras are calibrated with industrial accuracy and registered to a common coordinate system [23]. The authors only provide accuracy measures for static objects like cubes and cylinders, this however does not reflect on the reliability of the system to measure the dynamic shape of the foot. Samson *et al.* used this system to study foot roll-over under dynamic conditions [24], where the authors measure a lowest height data (LHD) picture of the foot at different time instances using the dynamic foot reconstruction system in [22]. This LHD image is created by projecting the available foot points onto to ground plane and assigning to them their corresponding height value. The authors performed an analysis of the data by looking at five reconstructions of the right foot of ten healthy patients. This type of analysis could be observed as a reliability/repeatability measures of their reconstruction system, it is therefore important to compare it to the results obtained with the system presented in this paper. Samson *et al.* partition the foot into seven regions of interest (ROI), and measure the average height of the foot at every reconstruction by looking at all the height points below 15 mm in every ROI. The authors also computed a projected surface as the percentage of the visible surface compared to the size of the ROI. As a final step, an intra-class coefficient (ICC) was computed between the five trials of every foot reconstruction, which averaged around 90% for all the measurements. Although this study presents important dynamic foot measurements with their corresponding reliability ranges, it will be shown in later sections that these results do not provide proper information to assess the accuracy or reliability of the used system as a dynamic foot reconstruction unit.

A recent attempt to dynamic foot reconstruction can be found in the work by Blenkinsopp *et al.*[25]. The system reconstructs the whole surface of the foot excluding the plantar surface. Reconstruction is achieved using 3 stereo camera pairs. The system requires the subject’s foot to be sprayed with water based face paint, creating a speckled black and white pattern. Although this system provides fast and accurate reconstructions, it also involves adding artifacts to the foot that might be undesirable in clinical environments.

A summary of the review presented above can be found in Table 1, where the experimental data available for every design is presented and partitioned in 2 categories, first experiments done with artificial surfaces, and second results obtained with real feet in both static and dynamic conditions. Details about these results and their comparisons to the system presented in this manuscript will be further elaborated in the sections to follow. The last column in Table 1 also explains the principal disadvantages of each system in terms of design and clinical usability.

**Table 1:** Summary of 4D reconstruction systems available in literature

**Method**	**Planar objects**	**Real feet**	**Main disadvantages**
	**Accuracy/Repeatibility**	**Static/Dynamic**	
Coudert *et al.*[13]	NA/NA	NA/NA	Technique requires spraying foot with paint or adding sock
Jezersek & Mazina [14]	0.2 mm/NA	0.4 mm/NA	The system requires several specialized camera-projector-mirror systems
Jezersek *et al.*[16]	0.5 mm/NA	NA/NA	Similar to Jezersek & Mazina, the system requires expensive specialized equipment
Kouchi *et al.*[17]	0.5 mm/NA	2.0 mm/NA	System only measures 4 cross-section areas of the foot, which need to be manually marked on the foot surface
Schmeltzpfenning *et al.*[18]	NA/NA	NA/NA	Camera-projector systems work sequentially thus reducing the acquisition frequency
Mochimaru *et al.*[20]	NA/NA	NA/NA	Matching random pattern is computationally expensive and can create unpredictable errors
Yoshida & Kouchi [21]	NA/NA	4.0 mm/NA	System uses one generic model for all foot reconstructions; shape deformations during walking cannot be fully accounted by the generic model
Liu *et al.*[22]	0.25 mm/NA	NA/NA	System provides good visual results, but lacks proper experiments with real feet
Blenkinsopp *et al.*[25]	NA/NA	NA/NA	Reconstruction is based on painted artifacts on the foot surface

For every available system, available experimental results are presented.

Given the analysis presented in this section, this paper presents a 4D Foot Reconstruction System (henceforth 4DFRS) that uses a single camera/projector pair to reconstruct the plantar surface of the foot in motion, along with the appropriate tests to show the potential use for the system in a clinical scenario.

## Methods

### System overview

**Figure 1:**
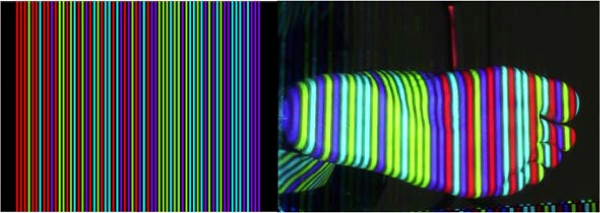
**CSL projector data.** Left: Light pattern used by the projector to illuminate the foot surface. Right: An example of a foot illuminated by the structured light pattern.

As presented in the previous sections, one of the principal challenges of reconstructing the foot is the lack of texture available in its surface. An effective solution in many reconstruction applications has been to create ‘artificial’ texture by projecting light onto the target surface. Although this light could be any random pattern desired, adding structure to the projection results in a simpler and more efficient solution. To this effect, Coded Structured Light (CSL) is used, whereby a coded sequence of colored stripes is implemented to facilitate detection [15]. The 4DFRS embraces this technique with a camera-projector set-up that implements a CSL technique. The 4DFRS embraces this technique with a camera-projector set-up that implements a CSL technique. Figure 1 shows the light pattern used with an example of a projection on the foot. The pattern of vertical stripes gets distorted by the presence of the foot surface, and the level of distortion is proportional to the foot shape. By analyzing the changes in the light pattern projected to the surface, the 3D shape of the foot can be estimated. Technical details about the formation of the CSL pattern can be found in the Additional file 1.

**Figure 2:**
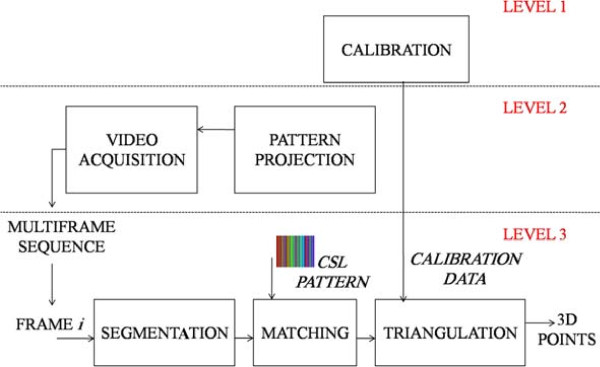
**4DFRS system diagram.** The diagram presents the different system stages as well as their inter-relations.

The diagram in Figure 2 shows the architecture of the 4DFRS and its three main modules. The first is the *calibration module* which is executed only when a physical change in the system occurs. The second is the *sequence acquisition module* which is executed to reconstruct a 4D model of a foot. The third is the *3D reconstruction module* which is executed for every video frame obtained during the acquisition process. Technical details of these modules are found in [15,26,27], as well as in the provided Additional file 1.

### System implementation and experiment design

**Figure 3:**
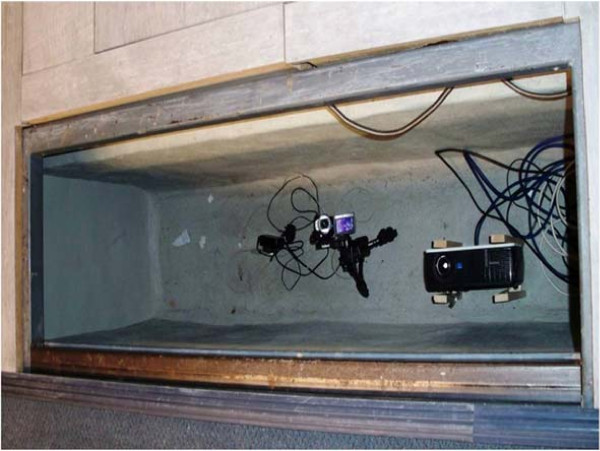
**4DFRS.** The system consists of a video camera and LCD projector, both embedded in a pit of a dedicated walkway. Picture imaged from above.

The 4DFRS prototype was set up in the gait laboratory of the Institute of Motion Analysis and Research (IMAR) of the University of Dundee. To minimize color distortions in the projected and observed stripe patterns, a three-chip camera (Panasonic®; HDC-H200 high-definition, with 1080 HD resolution) and 3-LCD projector (Sony®; VPL-EX4) were selected. The prototype was recessed in a dedicated walkway, covered by a transparent slab of toughened glass as a walking platform (Figure 3). The stand-off distance (system to target) was approximately 100 cm. The depth (range) of the reconstruction work-space is approximately 10 cm. We verified experimentally that the transparent platform did not alter the shape, position or color of the projected stripe patterns, i.e., the differences of reconstructions with and without the platform were smaller than the estimated reconstruction noise.

**Figure 4:**
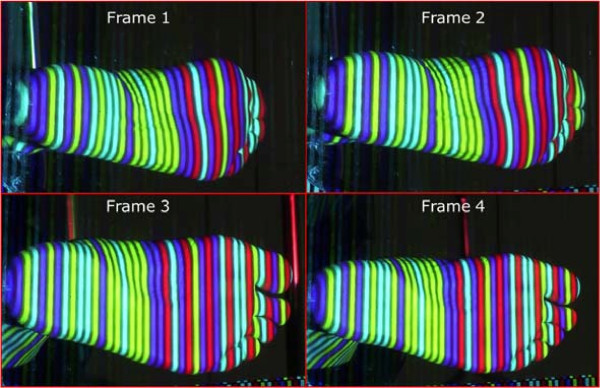
**Foot video sequence.** Four sample frames of a recorded step. On average, a step consists of 20 and up to 40 frames.

**Figure 5:**
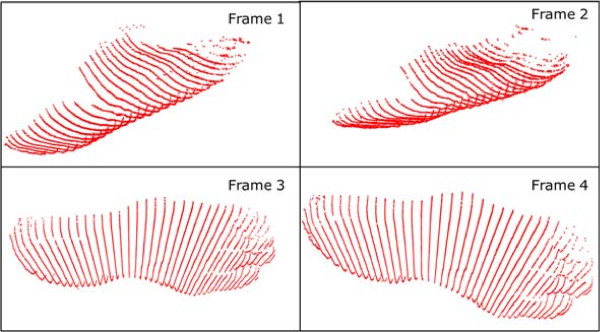
**Foot reconstructions 1.** The corresponding reconstructed shape (clouds of points) of the foot surface from the frames in Figure 4.

**Figure 6:**
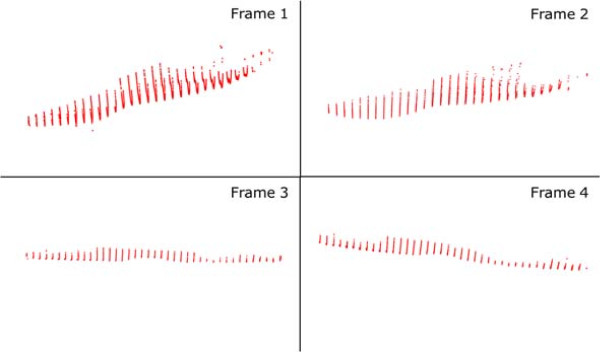
**Foot reconstructions 2.** Same reconstructions as in Figure 5, showing their corresponding orientations in space.

Figure 4 shows a portion of an input step recorded by the 4DFRS while Figures 5 and 6 display the corresponding reconstructed outputs; note that in these examples only four frames of the sequence are displayed (a common step covers 20 and up to 40 frames in some cases).

Experiments were carried out to assess the accuracy and repeatability of the prototype.

### Accuracy and repeatability

Accuracy refers to the similarity of a measured quantity and its true value, while repeatability refers to the level of agreement between several measurements taken under the same conditions. The accuracy of the system was tested first by imaging a plane made from rigid wood, with its surface painted matt grey. The dimensions of the plane were 30 cm × 15 cm, such dimensions compare well to the size of the foot. The plane was placed in the work space in ten different positions and orientations: zero inclination and elevation, zero inclination and 10 cm elevation, ±45° from the x-axis, ±45° from the y-axis, and ±45° from both the x- and y-axis. For each case, a 3D plane of the same size as the physical was fit to the reconstructed were computed as the distance of each point from the best-fit plane.

Repeatability quantifies error variations between different trials. Good repeatability is accounted to small variations in error among trials. Repeatability was assessed by capturing 20 images of a plane in two different positions, one lying on the ground and one slightly elevated (simulating two orientations of the foot surface taking a step), and computing reconstructions (the plane used was the same as in the accuracy experiments). The plane images were taken one straight after the other at full frame rate, thus the system was never switched off between acquisitions and the same calibration was used during the whole trial. The best-fit plane was estimated for each reconstruction, and mean absolute errors and standard deviations were calculated. The error was again defined as distance from the best-fit plane.

### Clinical repeatability

Clinical repeatability is a critical attribute for the current application. It is similar to repeatability as defined above, but is measured with real feet as done in a normal clinical session. The experiments presented in this section were therefore performed using real feet, in both static and dynamic situations. Twenty seven subjects were recruited. All participants were healthy with no previous surgery or known abnormality that could affect their gait pattern. Before the recruitment process took place, an application for ethical approval was submitted to the University of Dundee Research Ethics Committee and approval was granted in October, 2009. For each subject, four static and four dynamic sequences were acquired for both the left and right foot. Dynamic sequences were obtained at full frame rate (60 Hz). The subjects were asked to come back one week later to repeat the same measurements. Reconstructions of all the sequences were computed and a comparison performed between 3D foot shapes of each subject from day one and day two.

**Figure 7:**
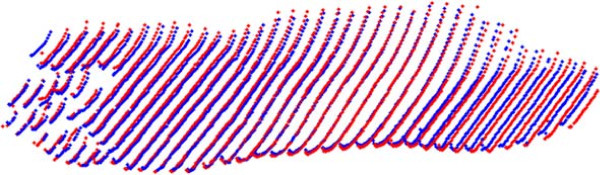
**Foot surface registration.** Registered static foot reconstructions using ICP (Blue foot corresponds to day 1 while red to day 2).

In the static case, an average of the four reconstructions was computed for each foot and compared with its corresponding average on the second day. In order to compare the two averages from different days, the surfaces were registered automatically using the Iterative Closest Point (ICP) algorithm [28,29]. The main idea behind ICP is to find the best registration between two surfaces, by minimizing the distance between closest points. In the first iteration, ICP finds a set of closest points in the reference surface, and it uses these relations to register the two frames. The algorithm iterates until the difference between closest points is smaller than some threshold. Although slow, ICP provides high accuracy in registering sets of points together. Figure 7 shows an example of two registered static surfaces taken on two different days.

In the dynamic case, comparing reconstructions from different sessions presents additional obstacles. The way a person takes a step varies over time, leading to different possible measurements. A first difference observed across trials is in the number of frames captured by 4DFRS for a single step. To compare 4D reconstructions in this case, five frames were selected manually from each reconstruction sequence, and errors computed for each frame as in the static reconstruction case. The five frames were selected using the following criteria: 

- Frame 1: The first frame of foot contact with the ground.

- Frame 2: Mid frame between Frames 1 and 3.

- Frame 3: Frame with foot on full ground contact. Several consecutive frames represent the foot in full contact with the ground, the mid-frame of these was selected.

- Frame 4: Mid frame between Frames 3 and 5.

- Frame 5: The last frame with the foot in contact with the ground.

All the images were analyzed and processed using **MATLAB**®;.

## Results

### Accuracy and repeatability

A summary of the accuracy results is presented in Table 2. Both mean absolute error and standard deviation were below 0.3 mm. To make sure that the reconstructed planes are of close size to physical plane imaged, a measure of surface coverage was obtained for every reconstruction and compared to the surface area of the plane. The average error of plane size was 0.2 mm. It is important to note that since the accuracy analysis was obtained with static planar surfaces, the result must be regarded as an experimental upper limit for the accuracy of the system when used with real feet in static and dynamic conditions.

**Table 2:** Results obtained in accuracy assessment

**Plane elevation (cm) - orientation (∘)**	**Mean (mm)**	**STD (mm)**
0 - 0	0.4	0.3
10 - 0	0.4	0.3
0 - 45 (x-axis)	0.3	0.3
0 - -45 (x-axis)	0.5	0.4
0 - 45 (y-axis)	0.5	0.4
0 - -45 (y-axis)	0.4	0.3
0 - 45 (x-axis) and 45 (y-axis)	0.4	0.3
0 - -45 (x-axis) and 45 (y-axis)	0.5	0.4
0 - 45 (x-axis) and -45 (y-axis)	0.3	0.2
0 - -45 (x-axis) and -45 (y-axis)	0.4	0.4
Mean (mm)	0.4	0.3
STD (mm)	0.3	0.1

A mean error and standard deviation is computed for every plane reconstruction. These values are obtained by comparing the reconstructions with their corresponding best fit planes.

Table 3 shows the results of the repeatability tests. The small inter-trial variation presented in the last row of Table 3 suggest good repeatability for the 4DFRS, but as in the accuracy case, these results can only be seen as upper limits to the system in real experiments.

**Table 3:** Results obtained in repeatability assessment

**Trial**	**Plane 1**		**Plane 2**	
	**Mean (mm)**	**STD (mm)**	**Mean (mm)**	**STD (mm)**
1	0.4	0.3	0.4	0.3
2	0.3	0.3	0.4	0.3
3	0.4	0.3	0.4	0.3
4	0.3	0.3	0.4	0.3
5	0.4	0.3	0.4	0.3
6	0.4	0.3	0.4	0.3
7	0.4	0.3	0.4	0.3
8	0.4	0.3	0.4	0.3
9	0.4	0.3	0.4	0.3
10	0.4	0.3	0.4	0.3
Mean (mm)	0.3	0.3	0.4	0.3
STD (mm)	0.003	0.001	0.003	0.002

A mean error and standard deviation is computed for every plane reconstruction. These values are obtained by comparing the reconstructions with their corresponding best fit planes. Since we want to measure the variations in the system computations, a total mean for all the means and STDs is computed along with its level of variation.

### Clinical repeatability

**Figure 8:**
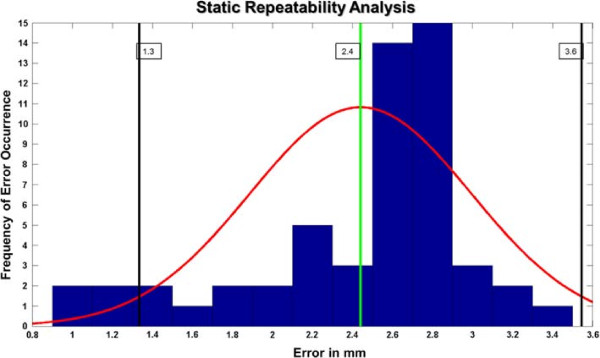
**Static repeatability.** Results of the static repeatability experiments. Main plot in blue is a histogram of error distribution. Green vertical line represents the mean error. Black lines show the interval contained between mean ± 2× standard deviation. Red curve shows the resulting normal distribution curve using the mean and standard deviation of the error data; this curve is intended at showing visually the similarity between the error and a Gaussian distribution.

Figure 8 summarizes the repeatability results for the static trials. The range of errors goes from 1.0 mm to 3.4 mm, with mean error of 2.4 mm and standard deviation 2.1 mm. Figure 8 presents a histogram of error distribution with a normal distribution curve plotted on top, this curve represents the shape of a possible Gaussian distribution coming using the mean and standard deviation of the data. Visual inspection shows the error to be close to normally distributed. The 95% confidence interval is also enclosed in the graph, which ranges from 1.3 mm to 3.6 mm.

**Figure 9:**
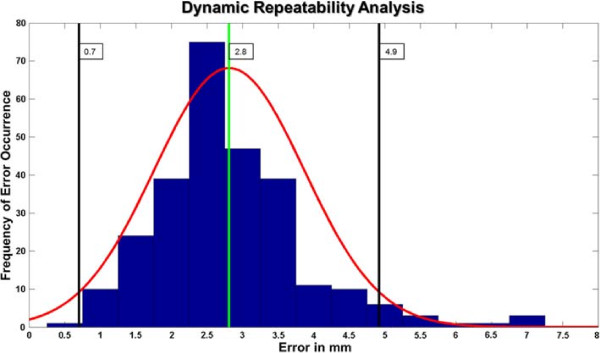
**Dynamic repeatability.** Results of the dynamic repeatability experiments. The components of the graph are like those in Figure 8.

In the dynamic case, the average difference between reconstructions in two trials, over the 27 subjects, was found to be 2.8 mm, with standard deviation 1.1 mm, while the whole error range varied between 0.5 mm to 7.0 mm. Figure 9 shows the error distribution for the dynamic repeatability results. The results in this case also resemble a Gaussian distribution. An average error was also computed for each of the five frames. The worst case average difference was of 3.4 mm, corresponding to frame four (heel off); this error was also the maximum in 49% of the subjects. The best-matching frame had a mean difference value of 2.3 mm and it came from the third frame (mid-stance); mid-stance corresponded to the best match in 38% of the subjects. A summary of the mean errors and standard deviations per frame can be found in Table 4.

**Table 4:** Clinical repeatability results per frame

**Plane**	**1**	**2**	**3**	**4**	**5**
**Mean (mm)**	3.0	2.5	2.3	3.4	2.8
**STD (mm)**	2.6	2.2	2.1	3.0	2.5

The mean and standard deviation of the error in comparison for the five dynamic foot frames selected.

**Figure 10:**
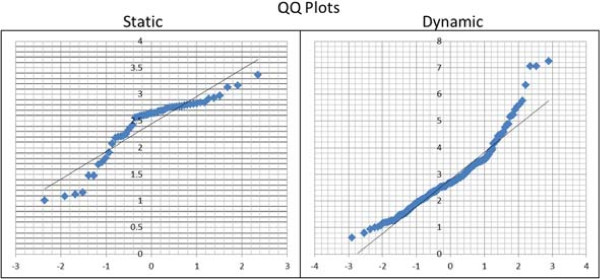
**Q-Q Plots for static a dynamic errors.** The Q-Q plots for error distributions. The proximity of the plot to a linear distribution suggests data close to normal. This is further validated by the correlation coefficients obtained.

The quantile-to-quantile (Q-Q) plots in Figure 10 further validated the normality hypothesis of the error distribution in both static and dynamic cases. Correlation coefficients for these plots were found to be 0.93 and 0.96 for the static and dynamic data respectively.

## Discussion

The aim of this research was to present a 3D dynamic foot reconstruction system, capable of obtaining plantar surface 3D models at high frame rates; the main drive was to present a system reliable enough to be used in clinical environments. While the clinical impact of the 4DFRS is not yet clear, encouraging results were recorded in tests on both planar objects and real feet. The 4DFRS proved to be accurate up to 0.5 mm when tested using planar objects, these results compare well with other systems in the literature. Many of these systems however, provide accuracy and repeatability results for limited scenarios, it is therefore difficult to produce a comprehensive comparison of these values with the experimental outcomes of the 4DFRS. Only the systems described in [14,16,17,22] provide system accuracy results using planar objects, while repeatabilty experiments are only documented in [14,17,21], for real feet under static conditions.

In order to better assess the 4DFRS when compared to the available designs, it is better to look at a combined comparison of numerical accuracy and repeatability results and design advantages/disadvantages. Table 1 in Section ‘Background’, presented a summary of the current techniques available in the literature, with their corresponding measures of accuracy and repeatability where available. In addition, the last column on Table 1 describes the main disadvantage of the design when compared to the current system.

In terms of design, the 4DFRS is superior to other systems in 4 main factors: 

- The system does not require adding artifacts (paint/socks/etc...) to the subject’s foot, which provides better convenience in clinical scenarios.

- The 4DFRS is built using a single off-the-shelf camera/projector system, it is therefore cheap and easy to build, without compromising on accuracy and reconstruction frequency.

- Due to the coded pattern used in the projections, reconstruction of the surface requires fewer computations and provides lower 3D errors.

- The 4DFRS has been tested with real feet in both static and dynamic conditions.

**Figure 11:**
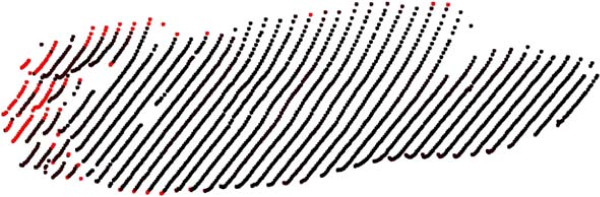
**ICP Registration error.** Results of the distribution of errors in registration plotted over the surface of one points cloud. The red points correspond to high errors (> 5 mm). As expected from calibration, large errors are mainly found at the peripherals of the surface.

In addition to design, the 4DFRS provides results for repeatability experiments under dynamic conditions. This type of analysis is not present in any other foot reconstruction scheme. Repeatability is an important measure, particularly when the system is to be used by clinicians and foot specialists. To analyze the repeatability errors, it is important to understand their source of occurrence. Part of the error comes from ICP; the presence of noise in the surface can limit the performance of the algorithm. Figure 11 shows one of the registered surfaces, with the red points corresponding to registration errors higher than 5.0 mm. These large errors are always present at the periphery of the reconstruction, a common factor that arises from lens distortion of the camera and/or projector [30]. The variable nature of walking, and manual selection of frames, i.e., frames selected for each configurations in the various sequences do not capture exactly the same instant of a walk, also account for the presence of high errors in the dynamic repeatability tests. These factors can explain some of the outliers outside the confidence interval in the dynamic experiments.

The experiments presented in this paper provide a bound of the repeatability of the 4DFRS in static and dynamic conditions. These results showed that the system is repeatable in the dynamic case, with most errors between 0.7 mm to 4.9 mm. It is difficult to specify whether these errors are tolerable enough for a foot reconstruction system, the quality of reconstruction will always be bound to the particular application of the system. The repeatability results show positive indications on the capabilities of the 4DFRS to reconstruct plantar foot shapes in dynamic conditions, and prompt future experiments to better qualify the system measurements. Results also compare favorably with the analysis of Maetzler *et al.*[31] and Ramanathan *et al.*[32] on the repeatability of different foot measurement systems.

Since the work presented by Samson *et al.* in [24] provides measurement data of the foot using the dynamic foot scanner the authors developed in [22,23], it is important to mention how their results compare to the data obtained by the 4DFRS. Although the data provided by Samson *et al.* presents some sort of clinical repeatability measure of the reconstruction system, it has two principal disadvantages. The first comes in the variables selected for measurement, mean height and projected surface. Mean height is calculated as the average of the distances from the ground plane to each of the foot points. Although each individual height measure provides information about the 3D nature of the point being looked at, the cumulative measure can be deceiving, since equal mean heights could come from differently shaped feet. The reliability/repeatability analysis of the 4DFRS is based on 3D point measurements between different trials and those comparisons are only done between points corresponding to the same plantar foot area. The projected surface measure can complement the mean height by analyzing the amount of foot surface seen at every ROI in every trial, but it still fails to provide a 3D shape measurement. A second disadvantages comes from the way the different trial reconstructions were acquired. Obtaining different trials per session is important to ensure reliable data, but it is not enough to assess the repeatability of the system. By analyzing the acquisition of several foot reconstructions at different time periods, proper clinical repeatability was measured for the 4DFRS.

Providing accurate dynamic foot measurements will further enhance the accuracy of applications clinicians and foot specialists are involved with in their daily duties. To provide such facilities, the 4DFRS was designed to be used in clinical environments, thus providing a scheme that reconstructs the plantar surface of the foot in a fast, accurate, and cheap manner, without involving the addition of markers in the subjects foot. Based on the literature presented in the Background section of this manuscript, no other system provides such a comprehensive approach. Given proper further experimental analyses of the system, the 4DFRS could provide better solutions to the clinical community and open additional applications in fields such as custom insole design, all without requiring higher compromises in terms of clinical usability.

In terms of limitations, the 4DFRS cannot provide scans of the entire foot and is only limited to the plantar surface. Reconstructing the surface in contact with the ground is always the central challenge of any foot scanner, it was therefore essential to solve this problem first before addressing the reconstruction of the remainder surface of the foot. Future enhancements of the 4DFRS will add complete foot reconstruction capabilities. Additional work needs to be done to ensure the system to be portable and easy to install. At the moment the system was mounted in a deep walkpit (100 cm). To ensure mobility of the system, it needs to be tested in a more compact configuration. The only factor that can limit compactness is the field of view of the camera and projector, a feature that can be easily adjusted with most commercially available hardware.

## Conclusion

This paper presented a novel design and implementation of a structured-light prototype system capable of reconstructing the shape of the plantar surface of the foot in motion, along with the system’s accuracy and repeatability experiments. The results obtained under dynamic conditions suggest good accuracy and repeatability of the system when compared to the available literature. Analysis of the system design also shows the 4DFRS improving on some of the limitations available in current systems. This final point comes from the fact that our system not only provides better estimates of dynamic repeatability, but also introduces a prototype that shows encouraging prospects for use in clinical trials.

Although the results presented in this paper provide a positive initial assessment of the 4DFRS, further analysis needs to be done to put the system in a more clinical context; this will require evaluation of different foot measurements, length, girth, foot contour shapes, and others. In that context, future work will involve the use of the 4DFRS in clinically involved situations, which will provide more insight into the role the system can play in foot analysis under dynamic conditions, as well as possible aid for therapeutic interventions such as custom footwear and insole design.

## Consent

Written informed consent was obtained from the patient for the publication of this report and any accompanying images.

## Competing interests

The authors declare that they have no competing interests.

## Authors’ contributions

AKT conducted the main research, design, and testing of the system. RJA provided support on the clinical aspects of the study and was the main party in securing funding. ET and JS supplied advice, as well as coding tools and testing tools for the computational side of the project. WW carried out the relevant statistical analysis. All authors read and approved the final manuscript.

## Supplementary Material

Additional file 1 Supplementary material. Click here for file
