# Supplementary material for: Dynamic 3D shape of the plantar surface of the foot using coded structured light: a technical report
Source: J Foot Ankle Res. 2014 Jan 23;7:5. doi: 10.1186/1757-1146-7-5 (PMC3903035; doi:10.1186/1757-1146-7-5)
Supplement: Additional file 1 — Supplementary material. [file 1757-1146-7-5-S1.pdf]

Supplementary Material

System Design

This section provides the reader with a more technical approach to how the 4DFRS was designed and implemented.

Calibration

Many camera calibration techniques are available in the computer vision field [1]. Techniques relevant to our application assume a perspective camera modelled by the well-known pinhole model [2]. A projector can be thought of as an inverse camera, and the same pinhole model applied [3]. Hence, the system model includes intrinsic parameters (focal length, image centre, aspect ratio of pixels for camera and projector) and extrinsic parameters (translation and rotation of the camera and projector with respect to each other).

The calibration target is composed of two perpendicular planes, each containing 20 equally sized squares, each 20 mm. The spacing between squares is also 20 mm. The origin of the global reference frame is at the bottom intersection of the two planes. The global co-ordinates of the corners of the 3D squares and the image co-ordinates of their corresponding image points are used to estimate the calibration parameters. We adopted the Faugeras-Toscani calibration algorithm in 4DFRS [4].

Projector parameters are calibrated after camera parameters. A grid pattern is projected onto the calibration pattern, in the same position used to calibrate the camera. The grid corners and their images (Figure 1) are used as calibration points. Taking into account the known geometry of the calibration object, and using the camera calibration parameters, the 3D locations of the grid intersections can be computed by triangulation. Once these are known, the Faugeras-Toscani algorithm can be used to estimate the calibration parameters of the projector [3].

Sequence acquisition

A comprehensive review of CSL techniques by Salvi *et al.* [5] shows that techniques using spatial neighborhood coding are the most suitable for dynamic surface reconstruction [6–10]. The 4DFRS uses the simple yet accurate approach by Pages *et al.* [3], i.e., spatial-coded/peak-based structured light. Figure 1 illustrates the pattern used in the foot reconstruction system. It consists of 64 colored stripes with black bands between each pair of consecutive stripes. The arrangement of stripes is based on a *De Bruijn* sequence of four colors and window property of three, meaning that any three consecutive stripes form a unique color sequence

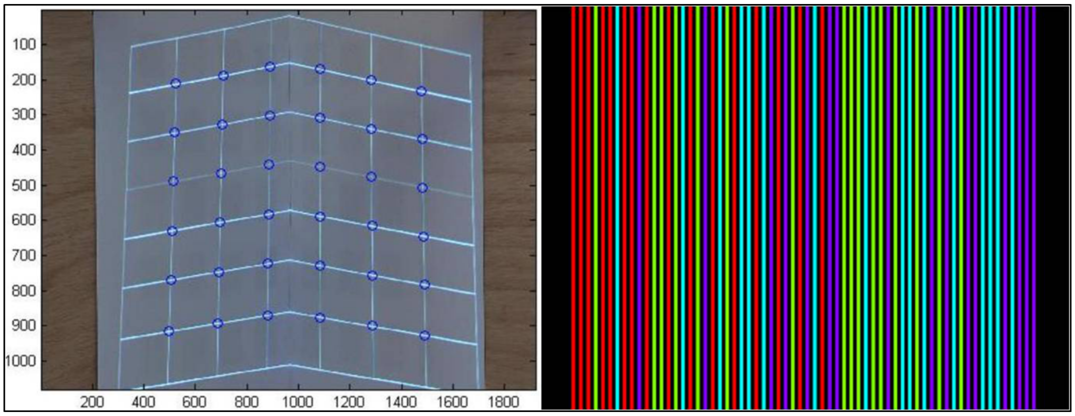

Figure 1: Left: Image of projection pattern superimposed onto the 2 planes of the calibration pattern. The blue circles mark the calibration points. Right: CSL Pattern.

within the pattern. This enables robust correspondence estimation. The pattern illuminates the foot surface taking a step as the video camera records a sequence of frames. These frames are then processed sequentially to reconstruct the shape of the foot surface. Since all the information needed for reconstruction is encoded in one pattern, it is possible to reconstruct the plantar surface of the foot at every image, and therefore obtain full camera frame rates.

3D reconstruction

Correspondences between the image stripes and the original pattern are calculated. This process occurs in two stages. Stage one consists of locating the centers of the colored stripes on the captured image; stage two compares the segmented stripes with the coded pattern in order to determine correspondences.

The center of each stripe is located row by row. At each pixel, a positive function $g(i)$ is computed, defined by:

$$g(i) = dR^2(i) + dG^2(i) + dB^2(i), \tag{1}$$

where i is the index of the pixel on a given row, R (resp. G, B) is the red (resp. green, blue) channel of the image, and d indicates the following filter applied to the monochromatic rows:

$$df(i) = \sum_{c=1}^{o/2}((f(i+c) - f(i-c)). \tag{2}$$

The parameter o is the spatial width of the filter. With an ideal top-hat signal, df is maximum and positive at the rising edge of the signal, zero at the center, and minimum and negative at the falling edge.

Consequently, the maxima of g identify the stripe edges. Stripe centers are located with subpixel accuracy as the normalized centroid of the non-black segments between two maxima [11].

Following Zhang *et al.* [10], the correspondence between image stripes and pattern stripes was solved using single-pass dynamic programming, a well-established approach in solving the correspondence problem in structured-light systems [3, 12, 13].

The 3D co-ordinates of surface points are calculated by triangulation. The back-projection ray through the center of the camera reference frame and a stripe pixel is intersected with the plane defined by the center of the projector reference frame and the pattern stripe corresponding to the pixel.

References

1. Salvi J, Armangue X, Batlle J: **A comparative review of camera calibrating methods with accuracy evaluation**. *Pattern Recognit* 2002, **35**:1617–1635.
2. Hartley R, Zisserman A: *Multiple view geometry in computer vision*. Cambridge, UK ; New York: Cambridge University Press, 2nd edition 2003.
3. Pages J, Salvi J, Collewet C, Forest J: **Optimised de Bruijn patterns for one-shot shape acquisition**. *Image Vis Comput* 2005, **23**:707–720.
4. Salvi J, Armangue X, Batlle J: **MatLab Camera Calibration Toolbox** 2002, [http://eia.udg.es/~qsalvi/recerca.html].
5. Salvi J, Fernandez S, Pribanic T, Llado X: **A State of the Art in Structured Light Patterns for Surface Profilometry**. *Pattern Recognit* 2010, **43**(8):2666–2680.
6. Boyer KL, Kak AC: **Color-encoded structured light for rapid active ranging**. *IEEE Trans. Pattern Anal. Mach. Intell.* 1987, **9**:14–28.
7. Maruyama M, Abe S: **Range Sensing by Projecting Multiple Slits with Random Cuts**. *IEEE Trans. Pattern Anal. Mach. Intell.* 1993, **15**:647–651.
8. Durdle NG, Thayyoor J, Raso VJ: **An improved structured light technique for surface reconstruction of the human trunk**. In *IEEE Canadian Conference on Electrical and Computer Engineering* 1998.
9. Monks TP, Carter JN, Shadle CH: **Colour-encoded structured light for digitisation of real-time 3D data**. *International Conference on Image Processing* 1992, :327–330.
10. Zhang L, Curless B, Seitz SM: **Rapid Shape Acquisition Using Color Structured Light and Multi-pass Dynamic Programming**. In *The 1st IEEE International Symposium on 3D Data Processing, Visualization, and Transmission* 2002:24–36.
11. Trucco E, Fisher RB, Fitzgibbon AW, Naidu DK: **Calibration, data consistency and model acquisition with laser stripers**. *International Journal of Computer Integrated Manufacturing* 1998, **11**:293–310.
12. Chen CS, Hung YP, Chiang CC, Wu JL: **Range data acquisition using color structured lighting and stereo vision**. *Image Vis Comput* 1997, **15**:445–456.
13. Ohta Y, Kanade T: **Stereo by Intra- and Inter-Scanline Search Using Dynamic Programming**. *IEEE Trans Pattern Anal Mach Intell* 1985, **PAMI-7**:139–154.
